# Predictors and clinical outcomes of post-coronary artery bypass grafting cerebrovascular strokes

**DOI:** 10.1186/s43044-022-00315-4

**Published:** 2022-10-18

**Authors:** Mohamed Laimoud, Mary Maghirang, Mosleh Alanazi, Shatha M. Al-Mutlaq, Suha A. Althibait, Boshra Alanazi, Munirah Alomran, Zohair Al Halees

**Affiliations:** 1 Cardiac Critical Care Department, King Faisal Specialist Hospital and Research Center, Riyadh, Saudi Arabia; 2 Critical Care Medicine Department, Cairo University, Cairo, Egypt; 3 Cardiac Surgery Department, King Faisal Specialist Hospital and Research Center, Riyadh, Saudi Arabia; 4 College of Medicine, Almaarefa University, Riyadh, Saudi Arabia

**Keywords:** CABG, Coronary artery bypass grafting, SOFA score, Acute cerebrovascular stroke, Chronic kidney disease, Carotid artery stenosis, Mechanical thrombectomy, Postoperative atrial fibrillation, Cardiopulmonary bypass, Mortality

## Abstract

**Background:**

Despite the improved medical and surgical managements, still there is a significant risk of developing acute cerebrovascular strokes after coronary artery bypass grafting (CABG). Our objectives were to study the immediate and long-term outcomes after CABG and to identify the possible predictors of post-CABG strokes.

**Results:**

Between January 2016 and August 2020, 410 adult patients, mostly males (82.2%), were retrospectively enrolled after CABG. Acute postoperative strokes occurred in 31 (7.5%) patients; of them, 30 (96.8%) patients had ischemic stroke, while 1 (3.2%) had hemorrhagic stroke. Mechanical thrombectomy was done in two cases. The patients who developed acute cerebral stroke had significantly higher admission (*p* = 0.02) and follow-up (*p* < 0.001) SOFA scores, higher arterial blood lactate level (*p* < 0.001), longer hospitalization (*p* < 0.001) and more hospital mortality (*p* < 0.001) compared with the patients who did not develop stroke. Kaplan–Meier curves for 5-year mortality showed increased risk in those patients with postoperative stroke (HR: 23.03; 95% CI: 6.10–86.92, *p* < 0.001). After multivariate regression, the predictors of early postoperative stroke were carotid artery stenosis (CAS), postoperative atrial fibrillation, cardiopulmonary bypass time, prior cerebral stroke, admission SOFA score and chronic kidney disease (CKD). The predictors of late cerebrovascular stroke were CAS, combined CABG and valve surgery, CKD, atrial fibrillation, prior stroke and HbA1c.

**Conclusions:**

The development of post-CABG acute cerebrovascular stroke is associated with longer hospitalization, multiple morbidities and increased mortality. Careful assessment and management of risk factors especially atrial fibrillation and carotid artery stenosis should be implemented to decrease this substantial complication after CABG.

## Background

The occurrence of cerebrovascular stroke after coronary revascularization is a substantial complication with a more frequency after CABG compared with percutaneous coronary interventions (PCI) [1, 2]. CABG is required in patients who are not suitable for PCI and still preferred in cases of significant coronary artery disease [3, 4]. Other indications of CABG include diabetes mellitus, left ventricular systolic dysfunction and rescue operation after failed trials of PCI [4, 5]. The development of postoperative cerebrovascular stroke is linked to prolonged hospitalization with high costs and increased hospital mortality [1, 2, 6]. Bucerius et al. [7] revealed the association between postoperative stroke and a sixfold increase in 30-day mortality. Due to advances in medications, percutaneous interventions and surgical techniques, the risk profiles of patients undergoing CABG change with increasing age and frequent comorbidities [8]. Also, atherosclerosis is a systemic vascular disease and patients subjected to CABG have high probabilities of significant atherosclerotic burden of different vessels including aorta, carotid and cranial arteries [9]. Our objectives were to study clinical outcomes of post-CABG acute cerebrovascular strokes and to identify the significant risk factors and potential predictors of postoperative stroke which will help to improve the patients' outcomes via adoption of new quality initiatives.

## Methods

### Study protocol

After getting our hospital ethical/research committee approval, we retrospectively enrolled all CABG patients (> 18 years) between 2016 and 2020 in this study. The data collected included demographic, perioperative clinical and laboratory variables, and the hospital outcomes. A follow-up was done to get the occurrence of a new stroke or mortality. The SOFA score was calculated after patient being admitted to ICU and 48 h later.

### Definitions of the variables

Acute ischemic stroke means an acute neurological manifestations persisting ≥ 24 h due to brain focal infarction in a well-defined vascular territory with exclusion of other possible etiologies [10]. Post-CABG early stroke means occurrence of stroke during the first 7 days after CABG, while late stroke occurs beyond the first 7 postoperative days [1]. Carotid artery stenosis (CAS) means narrowing of carotid lumen ≥ 50% by a plaque [11, 12]. Chronic kidney disease (CKD) means glomerular filtration rate (GFR) ˂ 60 mL/min/1.73 m^2^ for ≥ 3 months, according to the guidelines of Kidney Disease Improving Global Outcomes (KDIGO) group [13]. Acute kidney injury (AKI) means acute reduction in GFR according to the RIFLE criteria [14].

### Statistical analysis

The study results were presented using frequency with percentage for categorical data via the Chi-square (c2) test and using median with interquartile range (IQR) for quantitative data via the Mann–Whitney and Kruskal–Wallis tests. Data analysis was done using the statistical package for the Social Sciences (SPSS) version 26. Two-sided *P*-values < 0.05 were considered significant. After the univariate analysis, we used the significant variables for multivariate regression analysis to get the predictors of acute postoperative cerebrovascular stroke.

## Results

### Baseline characteristics

**Fig. 1:**
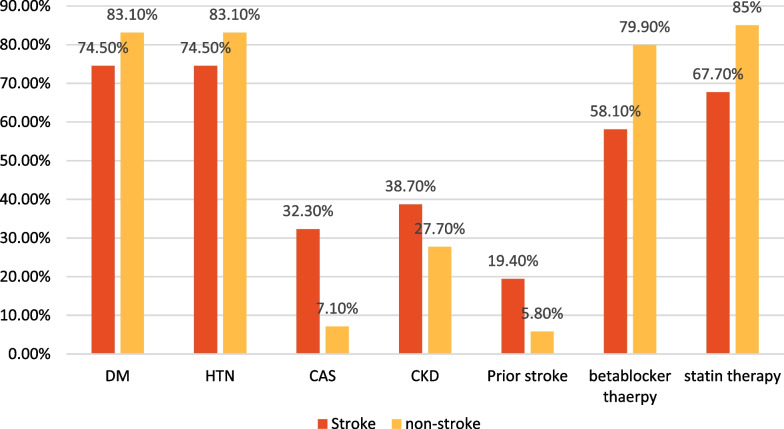
Preoperative variables of the enrolled patients

Our study enrolled 410 adult patients; of them, 31 (7.5%) patients developed acute cerebrovascular strokes after CABG. The patients with acute stroke had significantly more frequent prior strokes, carotid artery stenosis, CKD and prior cardiotomies while less frequent preoperative beta-blocker and statin therapies, and male gender compared to those who did not develop acute stroke (Table 1, Fig. 1).

**Table 1:** Characteristics and profiles of patients

Variables		All patients (410)	Stroke group (31,7.5%)	Non-stroke group (379,92.5%)	*P* value
Age (years)		60 (55–68)	66 (54–75)	60 (55–68)	0.06
Gender, male (*n*, %)		337 (82.2)	19 (61.3)	318 (83.9)	0.002
BMI (kg/m^2^)		28.05 (25.8–31.8)	28 (25–31.8)	28.1 (25.6–31.8)	0.88
Smoking (*n*, %)		159 (38.8)	11 (35.5)	148 (39.1)	0.69
Diabetes mellitus (*n*, %)		338 (82.4)	23 (74.5)	315 (83.1)	0.209
Hyperlipidemia (*n*, %)		245 (59.8)	19 (61.3)	226 (59.6)	0.85
Chronic Kidney disease (*n*, %)		117 (28.5)	12 (38.7)	105 (27.7)	0.041
Hypertension (*n*, %)		351 (85.6)	25 (80.6)	326 (86)	0.42
Bronchial asthma (*n*, %)		44 (10.7)	5 (16.1)	39 (10.3)	0.359
Hypothyroidism (*n*, %)		42 (10.2)	3 (9.7)	39 (10.3)	1
Hyperthyroidism (*n*, %)		3 (0.7)	0 (0)	3 (0.8)	1
Preoperative AF (*n*, %)		26 (6.3)	2 (6.5)	24 (6.3)	1
Malignancy (*n*, %)		26 (6.3)	2 (6.5)	24 (6.3)	1
Renal/liver transplant (*n*, %)		17 (4.1)	1 (3.2)	16 (4.2)	1
Prior stroke (*n*, %)		28 (6.8)	6 (19.4)	22 (5.8)	0.013
Prior TIAs (*n*, %)		24 (5.9)	3 (9.7)	21 (5.5)	0.41
Carotid artery stenosis (*n*, %)		37 (9)	10 (32.3)	27 (7.1)	< 0.001
Peripheral vascular disease (*n*, %)		44 (10.7)	3 (9.7)	41 (10.8)	1
Prior myocardial infarction (*n*, %)		257 (62.7)	24 (77.4)	233 (61.5)	0.07
Prior PCI (*n*, %)		137 (33.4)	10 (32.3)	127 (33.5)	0.88
Prior cardiac surgeries		27 (6.6)	7 (22.6)	20 (5.3)	0.002
Preoperative LVEF% (*n*, %)	More than 55%	141 (34.4)	10 (32.3)	131 (34.6)	0.052
	45–55%	106 (25.9)	4 (12.9)	102 (26.9)	
	35–45	88 (21.5)	6(19.4)	82 (21.6)	
	Less than 35%	75 (18.3)	11 (35.5)	64 (16.9)	
Preoperative drugs (*n*, %)	Beta-blockers	321 (78.3)	18 (58.1)	303 (79.9)	0.004
	Statins	343 (83.7)	21 (67.7)	322 (85)	0.013
	Fibrates	45 (11)	2 (6.5)	43 (11.3)	0.55
	ACEI/ARBs	187 (45.6)	10 (32.3)	177 (46.7)	0.12
	Steroids	18 (4.4)	1 (3.2)	17 (4.5)	1
	Immune suppression	11 (2.7)	1 (3.2)	10 (2.6)	0.58
Preoperative IABP (*n*, %)		15 (3.7)	3 (9.7)	12 (3.2)	0.09
Preoperative ECMO (*n*, %)		2 (0.5)	2 (6.5)	0 (0)	0.006

*BMI* body mass index, *AF* atrial fibrillation, *CAS* carotid artery stenosis, *PCI* percutaneous coronary intervention, *ACEI* angiotensin-converting enzyme inhibitors, *IABP* intra-aortic balloon pump, *LVEF* left ventricle ejection fraction, *TIAs* transient ischemic attacks, *ARBs* angiotensin receptor blockers, *ECMO* extracorporeal membrane oxygenation

### Laboratory variables

Preoperatively, the glycated hemoglobin (HbA1c) was 8.1% [7.4–9.1] vs. 7.1% [6.4–8.1], *p* = 0.005. The peak blood lactate level was 12.5 [7.6–14.7] vs. 5.1[3.7–8.5], *p* < 0.001, and the peak troponin level was 980 [474–1560] vs. 608 [387–1149], *p* = 0.004 in those with and without strokes, respectively. The peri-operative laboratory variables were summarized in Table 2.

**Table 2:** Laboratory variables

Variables	All patients	Stroke group	Non-stroke group	*P* value
Preoperative platelets (10ˆ^9^/L)	246 (202–308)	304 (229–352)	243 (201–304)	0.08
Preoperative hemoglobin (g/L)	131 (115–142)	118 (108–136)	131 (116–143)	0.052
Preoperative serum creatinine (umol/L)	90 (74–111)	85 (64–107)	90 (74–113)	0.1
Preoperative serum bilirubin (umol/L)	6.6 (4.6–11.1)	10.4 (6.6–17.4)	6.4 (4.6–10.4)	0.002
Preoperative INR	1 (1–1.1)	1.2 (1.1–1.2)	1 (1–1.1)	0.06
Preoperative fibrinogen (g/L)	3.2 (2.85–3.65)	3.43 (2.88–3.62)	3.19 (2.85–3.7)	0.72
Preoperative serum albumin (g/L)	39.8 (36.7–42.4)	39.1 (34.4–40.2)	40 (37–42.5)	0.052
HbA1c (%)	7.2 (6.4–8.2)	8.1 (7.4–9.1)	7.1 (6.4–8.1)	0.005
Peak lactate (mmol/L)	5.5 (3.8–8.9)	12.5 (7.6–14.7)	5.1 (3.7–8.5)	< 0.001
Peak troponin (ng/L)	634 (392–1180)	980 (474–1560)	608 (387–1149)	0.004
Hemoglobin day 1 (g/L)	98 (91–107)	90 (86–102)	98 (92–107)	0.001
Platelets day 1 (10ˆ^9^/L)	189 (139–232)	204 (106–275)	189 (140–231)	0.97
Serum albumin day 1(g/L)	36 (32.8–38.6)	34.2 (30–36.5)	36.3 (33–38.7)	0.006
Serum bilirubin day 1 (umol/L)	9.75 (7.2–15.6)	16.8 (10.3–28)	9.5 (7–14)	< 0.001
Serum creatinine day 1 (umol/L)	86.5 (74–116)	89 (76–108)	86 (74–119)	0.78
Hemoglobin day 3 (g/L)	91 (88–97)	89 (85–94)	92 (88–97)	0.027
Platelets day 3 (10ˆ^9^/L)	167 (127–211)	157 (83–223)	169 (127–211)	0.12
Serum albumin day 3(g/L)	36 (33–37.8)	34.1 (30.6–36.7)	36.3 (33.4–37.8)	0.042
Serum bilirubin day 3 (umol/L)	12.85 (9–19.9)	19.6 (13.8–36.3)	12.4 (8.8–19.6)	< 0.001

### Perioperative variables

**Fig. 2:**
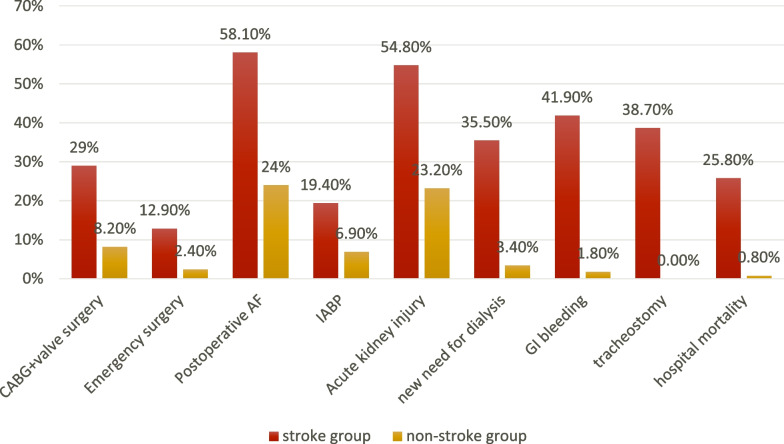
Postoperative variables of the studied patients

The patients with acute postoperative stroke had significantly longer cardiopulmonary bypass time, more frequent emergency surgeries, higher SOFA scores, higher frequencies of postoperative atrial fibrillation, ventricular arrhythmias, AKI, new need for dialysis, more inotropes days, more ventilator days, frequent tracheostomies, frequent ECMO support, frequent IABP support, frequent gastrointestinal bleeding, longer ICU stay, postoperative ward stay and increased hospital mortality compared to those who did not develop stroke, respectively (Fig. 2, Table 3).

**Table 3:** Perioperative variables and outcomes

Variables		All patients	Stroke group	Non-stroke group	*P* value
Type of surgery	Isolated CABG (*n*, %)	370 (90.2)	22 (71)	348 (91.8)	0.001
	CABG plus valve surgery (*n*, %)	40 (9.8)	9 (29)	31 (8.2)	
Cardiopulmonary bypass (minutes)		100 (77–126)	102 (90.5–157.5)	99 (76–125)	0.034
Aortic clamping time (minutes)		58 (43–83)	69 (46–84.5)	57 (43–83)	0.351
Approach	Sternotomy (*n*, %)	398 (97.1)	31 (100)	367 (96.8)	0.612
	Minimally invasive (n, %)	12(2.9)	0	12 (3.2)	
Urgency of surgery	Elective (*n*, %)	397 (96.8)	27 (87.1)	370 (97.6)	0.012
	Emergent (*n*, %)	13 (3.2)	4 (12.9)	9 (2.4)	
Initial SOFA score		2 (1–3)	3 (2–4)	2 (1–3)	0.02
SOFA at 48 h		1 (0–2)	5 (1–7)	1 (0–2)	< 0.001
Postoperative atrial fibrillation (n, %)		109 (26.6)	18 (58.1)	91 (24)	< 0.001
Ventricular arrhythmias (*n*, %)		24 (5.9)	6 (19.4)	18 (4.7)	0.006
Intra-cardiac thrombi (*n*, %)		7 (1.7)	2 (6.5)	5 (1.3)	0.09
Acute kidney injury (*n*, %)		105 (25.6)	17 (54.8)	88 (23.2)	< 0.001
New need for hemodialysis (*n*, %)		24 (5.9)	11 (35.5)	13 (3.4)	< 0.001
Exploration for bleeding (*n*, %)		27 (6.6)	4 (12.9)	23 (6.1)	0.137
Gastrointestinal bleeding (*n*, %)		20 (4.9)	13 (41.9)	7 (1.8)	< 0.001
Sternotomy wound infection (*n*, %)		53 (12.9)	7 (22.6)	46 (12.1)	0.1
Wound debridement (*n*, %)		37 (9)	7 (22.6)	30 (7.9)	0.014
ICU days		4 (3–6)	11 (6–42)	4 (3–5)	< 0.001
Inotropes days		2 (1–2)	6 (2–10)	1 (1–2)	< 0.001
Ventilator days		1 (1–2)	10 (2–32)	1 (1–2)	< 0.001
Post-ICU ward days		5 (3–8)	11 (2–57)	5 (3–7)	0.015
Postoperative ECMO		8 (2)	4 (12.9)	4 (1.1)	0.002
ECMO days		9 (6–13)	9 (6–13)	9.5 (7–13)	1
Postoperative IABP (*n*, %)		32 (7.8)	6 (19.4)	26 (6.9)	0.025
IABP days		3 (2–4)	3 (2–3)	3 (2–4)	0.644
Tracheostomy (*n*, %)		12 (2.9)	12 (38.7)	0 (0)	< 0.001
Hospital mortality (*n*, %)		11 (2.7)	8 (25.8)	3 (0.8)	< 0.001
Discharge with tube feeding (*n*, %)		13 (3.2)	12 (38.7)	1 (0.3)	< 0.001
Discharge with tracheostomy breathing (*n*, %)		10 (2.4)	10 (32.3)	0 (0)	< 0.001
Stroke during follow-up (*n*, %)		15 (3.7)	0 (0)	15 (4)	0.61
Mortality during follow-up (*n*, %)		28 (6.8)	2 (6.5)	26 (6.9)	1

**Fig. 3:**
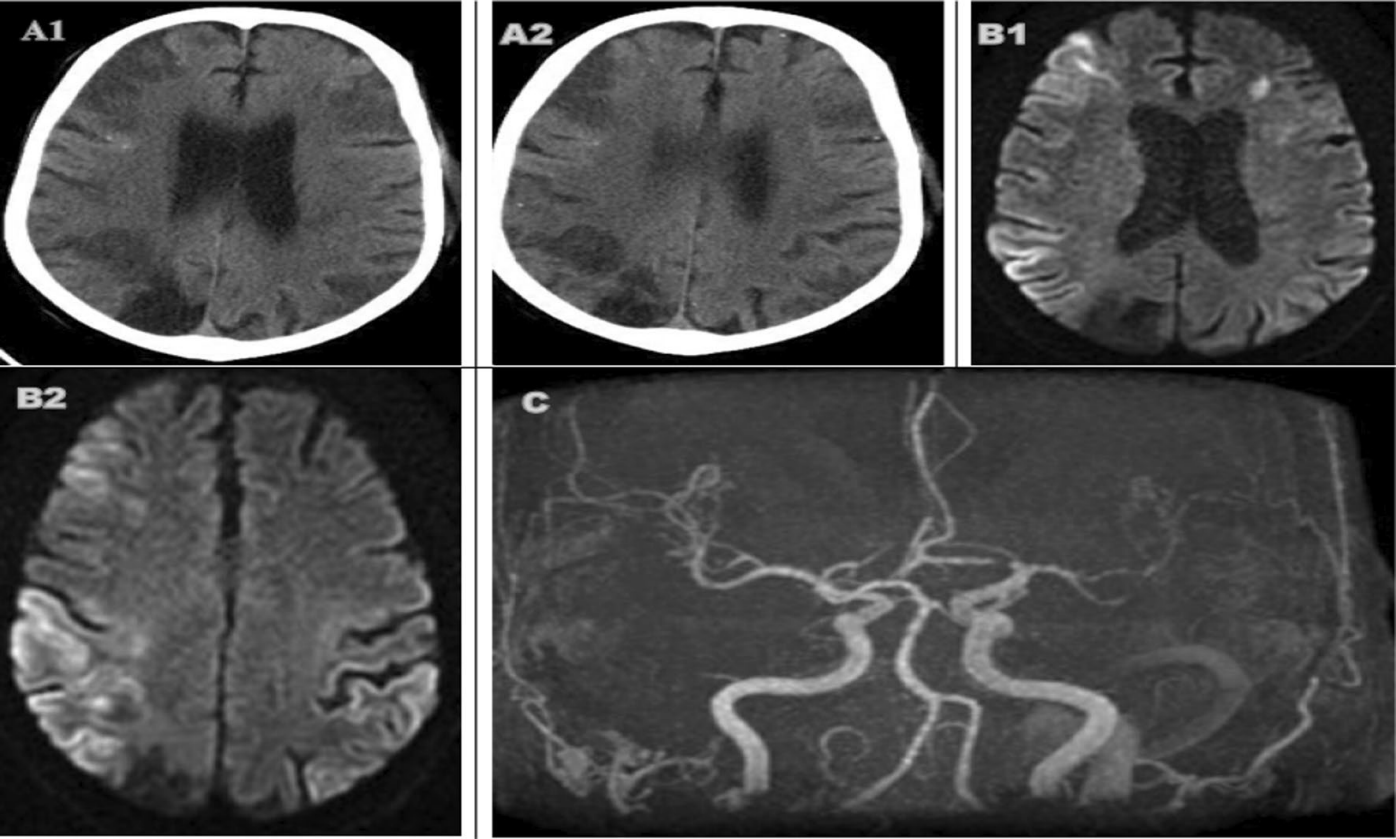
A 64-years-old man with old right occipital old stroke and multiple scattered calcific emboli, presented with delayed neurological recovery after CABG. CT brain (**A1**, **A2**) showed acute hypo-densities in bilateral fronto-parietal and right occipito-temporal regions representing watershed infarctions. MRI (**B1**, **B2**) was done after 6 days and showed external and internal watershed infarctions involving bilateral cortical and subcortical areas. MR angiography (**C**) demonstrates multifocal moderate-to-severe bilateral middle cerebral arteries stenosis associated with paucity of distal vessels with few collaterals' formation

**Fig. 4:**
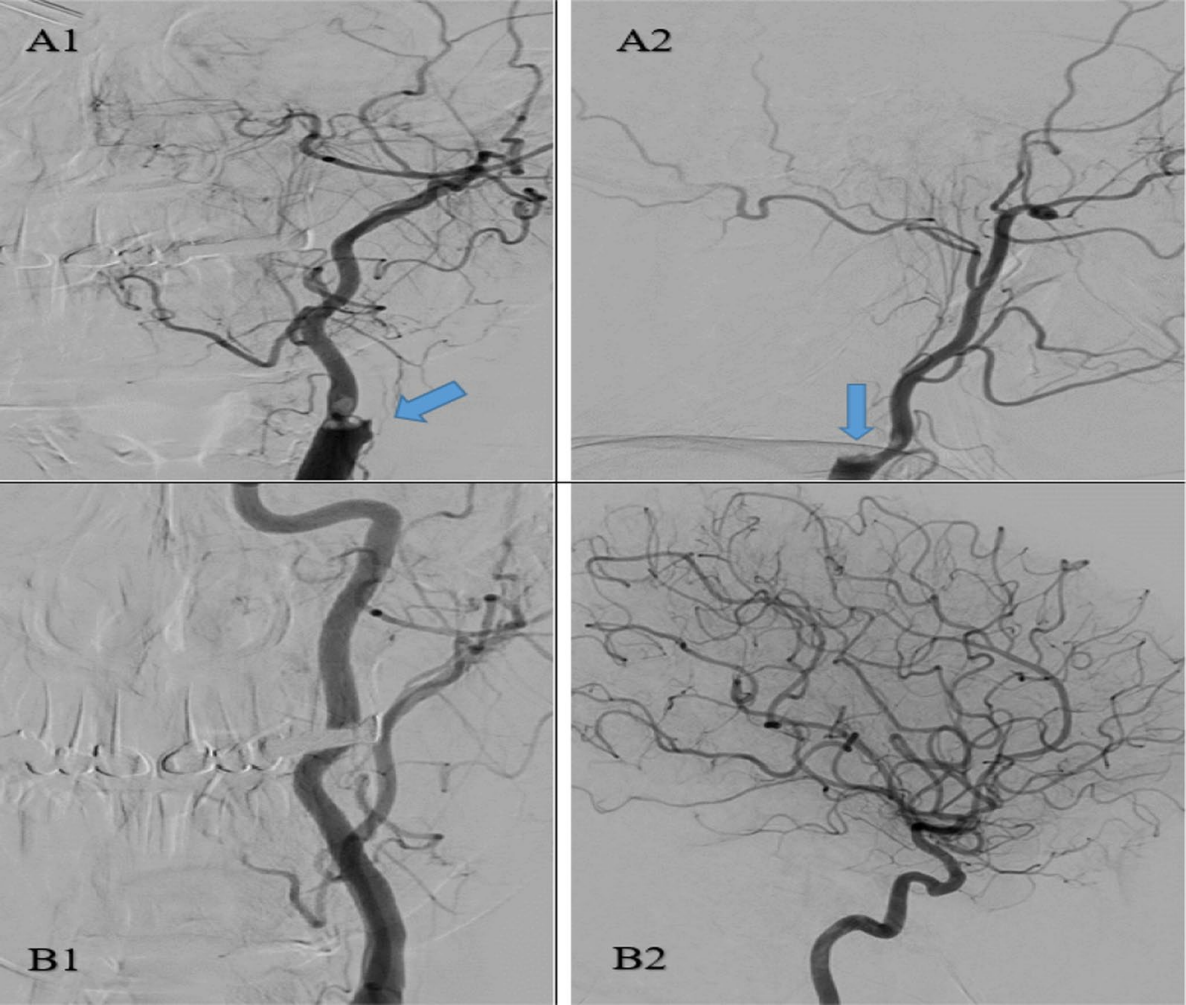
A 53 years-old-woman with atrial fibrillation that developed postoperative acute right-sided hemiparesis. Left carotid angiogram showed a filling defect (consistent with an embolic clot) completely occluding the internal carotid artery and partially occluding the origin of external carotid artery (**A1** & **A2**). Complete recanalization of both internal and external carotid arteries was achieved after mechanical thrombectomy (**B1** & **B2**)

According to brain imaging studies, there were territorial infarcts and watershed infarcts. Mechanical thrombectomy was done in two cases with large vessels occlusion (Figs. 3 and 4).

**Fig. 5:**
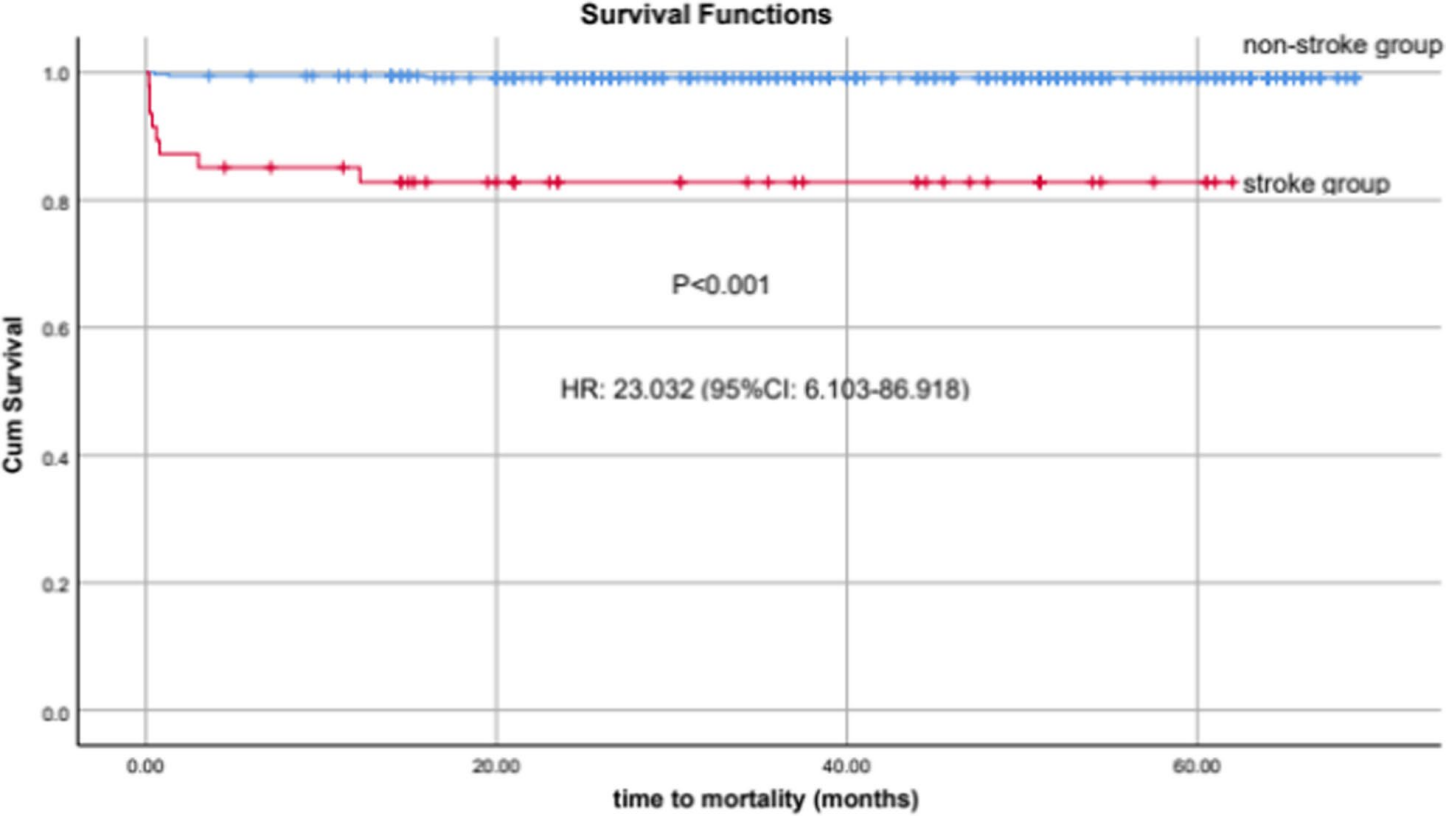
Kaplan–Meier curves of survival for 5-years follow-up

Kaplan–Meier curves of mortality during 5-years follow-up showed increased risk in those patients with postoperative stroke (HR: 23.032; 95% CI: 6.103–86.918, *p* < 0.001) (Fig. 5).

### Predictors of post-CABG strokes

Regression analysis was done to detect the predictors of post-CABG cerebrovascular stroke. Early stroke was independently predicted with CAS, CPB time, initial SOFA score, prior stroke, postoperative AF and CKD. Late cerebrovascular stroke was independently predicted with CAS, AF, prior stroke, CABG plus valve surgery, HbA1c and CKD (Table 4).

**Table 4:** Multivariate regression analysis

Studied variables	Odds ratio	95% CI	*P* value
*Early stroke*			
Carotid artery stenosis	8.223	2.92–23.1	< 0.001
Cardiopulmonary bypass time	5.64	1.97–16.11	0.001
Postoperative AF	3.014	1.158–7.84	0.024
Initial SOFA score	1.643	1.194–2.26	0.002
Prior stroke	4.412	1.286–15.14	0.018
Chronic kidney disease	1.28	1.05–3.75	0.017
*Late stroke*			
Carotid artery stenosis	9.38	3.72–23.66	< 0.001
CABG plus valve surgery	7.44	2.75–20.145	< 0.001
Chronic kidney disease	2.33	1.056–5.165	0.036
HbA1c	1.431	1.071–1.914	0.016
Prior stroke	3.725	1.179–11.768	0.025
Atrial fibrillation	2.35	1.025–5.41	0.016

## Discussion

Our study reported that 7.5% of CABG patients developed acute postoperative cerebrovascular strokes, and the occurrence of stroke was associated with longer hospitalization, many morbidities and increased mortality. Moreover, Kaplan–Meier curves of 5-year survival showed increased mortality in those patients with postoperative stroke (HR: 23.03; 95% CI: 6.10–86.92, *p* < 0.001). Many studies reported different incidences and mortality after CABG due to differences with the study designs, studied populations and surgical techniques [7, 9, 15, 16].

Bucerius et al. [7] studied a total of 16,184 patients and reported that 1.9% of isolated CABG patients and 7.4% of combined valve and CABG surgery patients developed acute postoperative stroke and confirmed that postoperative stroke was linked to a sixfold increase in 30-day mortality. Cao et al. [9] studied 430 CABG patients and reported an incidence of acute stroke of 7.4%. Dacey et al. [15] studied 35,733 CABG patients and reported occurrence of acute stroke in 1.61% with increased mortality and decreased 5-year survival among patients with physical limitations. Tarakji et al. [16] retrospectively studied 45,432 CABG patients with a 1.6% incidence of acute stroke and reported the association of stroke with worse many outcomes and longer hospitalization compared with the non-stroke group.

According to our results, the inotropic support, arrhythmias, postoperative IABP and ECMO support and multi-organ dysfunctions were significantly more frequent in the stroke group compared with the non-stroke group. Gaudino et al. [1] reported that thromboembolism and hypoperfusion were the causes of intra-operative stroke, while early postoperative stroke occurs due to unstable hemodynamics and arrhythmias. Post-cardiotomy low cardiac output syndrome (LCOS) represents a life-threatening state with severe reduction in cardiac systolic function and cardiac index resulting in impaired global tissue perfusion, multi-organ dysfunction and increased mortality [17, 18]. Algarni et al. [18] studied 25,176 CABG patients and reported that LCOS was predicted with emergency surgery, re-operative CABG, female gender and severe left ventricular systolic dysfunction. Our results showed significantly increased frequencies of AKI and dialysis in the stroke group which may be related to the postoperative renal hypoperfusion and increased inotropic support ± mechanical circulatory support. We used postoperative arterial blood lactate level to detect tissue hypoperfusion, and the patients with acute cerebral stroke showed progressive hyperlactatemia. Post-cardiotomy hyperlactatemia has been linked to increased mortality and different morbidities [19–21]. We used the SOFA scoring of the studied patients and for follow-up of organ functions after 48 h. The SOFA score was used in many studies and proved a high performance in different patients' groups including post-cardiotomies and patients on ECMO support [22, 23]. The SOFA score was associated with the early postoperative stroke in our regression analysis.

Perioperative cerebral embolism may occur due to cardiac, aortic, carotid, or cardiopulmonary bypass circuit sources. In this study, CPB time was significantly prolonged in the acute cerebral stroke group and was a predictor of early stroke. J. Bucerius et al. [7] reported that CPB time ≥ 120 min was a predictor of stroke and also reported the small stroke incidence with beating heart surgery. Aortic cannulation, application or removal of clamping and proximal graft anastomoses are important embolic sources during CABG [24, 25]. Ascending aorta atherosclerosis was documented in ≥ 50% of CABG patients and dislodgment of atheromatous debris or calcium can occur during application or removal of cross-clamping [25, 26]. It was reported that postoperative stroke occurred in 8.7% and 1.8% in patients with and without ascending aorta atherosclerosis, respectively [27]. There is a synergistic relation between cerebral embolism and hypoperfusion during CABG especially with the presence of carotid atherosclerosis resulting in watershed infarcts [28, 29]. Watershed infarcts may be cortical/external between the cortical branches of cerebral arteries or internal in the white matter between cortical and perforator branches of cerebral arteries [29]. Gottesman et al. [28] showed the association between watershed infarcts and intraoperative hypotension and reported the increased mortality with these infarcts. Also that study reported that MRI (48%) was better than CT scans (22%) for detection of watershed infarcts.

Carotid artery stenosis (CAS) was associated with both early and late postoperative strokes in our study. Raffa et al. [30] recently reported the increased risk of post-cardiotomy stroke with presence of CAS regardless of the degree of stenosis. Naylor et al. [31] reported that CAS was a predictor of postoperative stroke and the risk positively correlated with increased severity of CAS. Stamou et al. [32] retrospectively studied 16,528 CABG patients and reported that the stroke predictors included CAS, CKD, DM, prior cerebrovascular stroke, left ventricular systolic dysfunction, atrial fibrillation and age more than 75 years.

Our regression analysis showed that presence of previous cerebrovascular stroke was a predictor of both early and late new postoperative stroke and this finding agreed with other previous studies [7, 32]. So proper preoperative assessment for recurrent strokes should be done including carotids and ascending aorta evaluation.

Postoperative atrial fibrillation (POAF) occurred in 26.6% of our studied patients and was a predictor of early post-CABG stroke in the multivariate analysis. Moreover, atrial fibrillation was a predictor of late post-CABG stroke. POAF was reported in about 25% of isolated CABG patients and 50% after combined CABG and valve cardiotomies [33, 34]. POAF was previously considered as transient post-cardiotomy arrhythmia, but it was linked to prolonged hospitalization, readmissions and increased mortality [35, 36].

Lahtinen et al. [34] studied 2630 CABG patients and reported that 36.5% of the patients with postoperative strokes had experienced attacks of POAF. Despite POAF was related to local inflammation with atriotomy incisions and pericardial disruption or systemic inflammation with cardiopulmonary bypass and catecholamine infusions, there are patients’ factors that make POAF persistent like underling cardiac dysfunction. Recently, POAF was reported as a predisposing factor to late stroke during follow-up after CABG [35, 37]. Compared with the non-stroke group, our results showed that preoperative use of beta-blockers and statin therapies was significantly less frequent, while postoperative inotropes therapy was more frequent in the acute cerebral stroke group. Peri-operative use of beta-blockers was recommended as a class I according to European Associations of Cardiothoracic Surgeons (EACTS) and the European Society of Cardiology (ESC) guidelines [38]. Perioperative β-blockers therapy, unless contraindicated, was recommended as a class I, while amiodarone was given a class IIa recommendation according to the European Association of Cardiothoracic Anesthetists (EACTA) and the Society of Cardiovascular Anesthesiologists (SCA) guidelines [39].

Chronic kidney disease was a significant variable associated with both early and late cerebrovascular strokes after CABG in our regression analysis. Safaie et al. [40] reported the increased mortality and frequent morbidities after CABG in patients with CKD. Li et al. [41] reported the association between CKD and 30 days’ mortality and multiple outcomes including acute postoperative stroke.

Despite our results showed insignificant frequencies of diabetes mellitus (DM) between patients with and without acute postoperative strokes, the glycated hemoglobin (HbA1c) levels were significantly different between both groups reflecting a difference in preoperative blood sugar control. The HbA1c was a predictor of late cerebrovascular stroke after CABG in our regression analysis. Nyström et al. [42] studied 53,820 CABG patients and reported the higher risk of cerebrovascular strokes in patients with DM during follow-up.

Chronic hyperglycemia was associated with increased cardiovascular and cerebrovascular risks due to endothelial dysfunction, oxidative stress and enhanced atherosclerosis. The atherosclerotic burden with DM was proved in different studies, and the risks associated with DM were related to the control of blood glucose level [43–45]. Recently, Gao et al. [46] conducted the CARE- II study and reported the extensive carotid atheromas with significant calcifications and lipid-rich necrotic cores in patients with DM.

Since the use of thrombolysis during peri-operative period is contraindicated and strokes were linked to increased mortality, mechanical cerebral thrombectomy has been an emergent procedure to improve the neurological outcomes [47, 48]. The experience of postoperative cerebral thrombectomy is limited to few case reports [49, 50]. In our heart center, we decreased the use of narcotic analgesia and deep sedation immediately after cardiac surgery for close neurological assessment and early detection of any focal sign of neurological impairment. In case of presumed stroke, stroke code is activated and brain imaging with perfusion study will be done for possibility of mechanical thrombectomy in case of large vessel occlusion. Cerebral thrombectomy is considered in case of ischemic stroke with proximal large artery occlusion within 6 h of manifestations and it may be extended to 24 h according to CT perfusion data [48, 51]. Early detection of acute stroke is particularly a crucial step in proper management and the presence of established easy referral pathway to the neurosciences department allowed us to use the stroke code after cardiotomy and providing the mechanical thrombectomy procedure for the proper candidates.

## Conclusions

The development of post-CABG acute cerebrovascular stroke is associated with longer hospitalization, multiple morbidities and increased mortality. Careful assessment and management of risk factors especially atrial fibrillation and carotid artery stenosis should be implemented to decrease this substantial complication after CABG.

## Study limitations

The study was a single-center retrospective analysis. We could not assess the extent of preoperative aortic atherosclerosis and the blood sugar control data during hospital perioperative stay.

## Data Availability

The data of the study are available with the corresponding author.

## Abbreviations

SOFA: Sequential organ failure assessment
POAF: Postoperative atrial fibrillation
CABG: Coronary artery bypass grafting
DM: Diabetes mellitus
CKD: Chronic kidney disease
CT: Computed tomography
AF: Atrial fibrillation
CAS: Carotid artery stenosis
OR: Odds ratio
CPB: Cardiopulmonary bypass
AKI: Acute kidney injury
CI: Confidence interval
TIAs: Transient ischemic attacks
MRI: Magnetic resonance imaging
LVEF: Left ventricular ejection fraction
