# Correction: Predictors and clinical outcomes of post-coronary artery bypass grafting cerebrovascular strokes

**DOI:** 10.1186/s43044-022-00318-1

**Published:** 2022-11-08

**Authors:** Mohamed Laimoud, Mary Maghirang, Mosleh Alanazi, Shatha M. Al-Mutlaq, Suha A. Althibait, Boshra Alanazi, Munirah Alomran, Zohair Al Halees

**Affiliations:** 1 Cardiac Critical Care Department, King Faisal Specialist Hospital and Research Center, Riyadh, Saudi Arabia; 2 Critical Care Medicine Department, Cairo University, Cairo, Egypt; 3 Cardiac Surgery Department, King Faisal Specialist Hospital and Research Center, Riyadh, Saudi Arabia; 4 College of Medicine, Almaarefa University, Riyadh, Saudi Arabia

**Correction: The Egyptian Heart Journal (2022) 74:76 (2022)** 10.1186/s43044-022-00315-4

Following publication of the original article [1], the authors noticed two amendments to be made to the declarations section.

## **Consent for publication**

For the 2 patients mentioned in Figs. 3 and 4, we got informed consents.

## **Acknowledgements**

The abstract was presented in Acute Cardiovascular Care association congress (ACVC 2022) of the European Society of Cardiology, and it was published in the European Heart Journal supplement. https://academic.oup.com/ehjacc/article/11/Supplement_1/zuac041.048/6576944.
